# Svalbamides A and B, Pyrrolidinone-Bearing Lipodipeptides from Arctic *Paenibacillus* sp.

**DOI:** 10.3390/md19040229

**Published:** 2021-04-17

**Authors:** Young Eun Du, Eun Seo Bae, Yeonjung Lim, Jang-Cheon Cho, Sang-Jip Nam, Jongheon Shin, Sang Kook Lee, Seung-Il Nam, Dong-Chan Oh

**Affiliations:** 1 Natural Products Research Institute, College of Pharmacy, Seoul National University, Seoul 08826, Korea; dye0302@snu.ac.kr (Y.E.D.); ddol1289@snu.ac.kr (E.S.B.); shinj@snu.ac.kr (J.S.); sklee61@snu.ac.kr (S.K.L.); 2 Department of Biological Sciences, Inha University, Incheon 22212, Korea; yj_lim@inha.edu (Y.L.); chojc@inha.ac.kr (J.-C.C.); 3 Department of Chemistry and Nanoscience, Ewha Womans University, Seoul 03760, Korea; sjnam@ewha.ac.kr; 4 Korea Polar Research Institute, Incheon 21990, Korea; sinam@kopri.re.kr

**Keywords:** *Paenibacillus*, Arctic, Svalbard, Marfey’s method, DP4 calculation, quinone reductase, lipopeptide, 3-amino-2-pyrrolidinone

## Abstract

Two new secondary metabolites, svalbamides A (**1**) and B (**2**), were isolated from a culture extract of *Paenibacillus* sp. SVB7 that was isolated from surface sediment from a core (HH17-1085) taken in the Svalbard archipelago in the Arctic Ocean. The combinational analysis of HR-MS and NMR spectroscopic data revealed the structures of **1** and **2** as being lipopeptides bearing 3-amino-2-pyrrolidinone, d-valine, and 3-hydroxy-8-methyldecanoic acid. The absolute configurations of the amino acid residues in svalbamides A and B were determined using the advanced Marfey’s method, in which the hydrolysates of **1** and **2** were derivatized with l- and d- forms of 1-fluoro-2,4-dinitrophenyl-5-alanine amide (FDAA). The absolute configurations of **1** and **2** were completely assigned by deducing the stereochemistry of 3-hydroxy-8-methyldecanoic acid based on DP4 calculations. Svalbamides A and B induced quinone reductase activity in Hepa1c1c7 murine hepatoma cells, indicating that they represent chemotypes with a potential for functioning as chemopreventive agents.

## 1. Introduction

Marine habitats were generally recognized as extreme environments exposing organisms to conditions of high salt, high pressure, and hypoxia, forcing them to develop unique physiologies in comparison to their terrestrial counterparts. Among the marine organisms, bacteria have always contributed significantly as a source for the discovery of new marine natural products, with 232 new compounds found in 2019 alone [1]. However, most (62.5%) of the new marine-bacterial molecules were derived from the single genus *Streptomyces*. We also reported the dimeric benz[*a*]anthracene thioethers donghaesulfins A and B, and rearranged angucyclinones donghaecyclinones A–C from marine-derived *Streptomyces* sp. SUD119 in 2019 and 2020, respectively [2,3]. Even though *Streptomyces* is chemically prolific and still provides numerous new bioactive compounds, the desire for compounds with greater structural diversity has brought about chemical investigations of a wider diversity of bacteria that extend beyond conventional phylogenetically biased chemical studies. The chemical examination of bacteria inhabiting the Arctic Ocean—a more extreme habitat than tropical or subtropical oceans that remains poorly investigated—represents a promising strategy for the discovery of new bioactive molecules.

**Figure 1:**
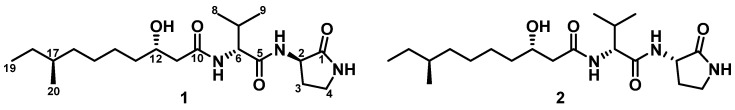
The structures of svalbamides A (**1**) and B (**2**).

In our continuing efforts to search for new bioactive microbial compounds from extreme marine environments, we explored the chemistry of bacterial strains from the Arctic Ocean. Our initial chemical profiling of Arctic strains led to the discovery of articoside and C-1027 chromophore-V—two new benzoxazine-bearing compounds that inhibit *Candida albicans* isocitrate lyase—from *Streptomyces* sp. ART5 collected from the East Siberian continental margin [4]. In this study, we diversified the phylogeny of bacteria for chemical analysis and focused on non-*Streptomyces* bacterial strains inhabiting the Arctic Ocean. The *Paenibacillus* sp. SVB7 strain was isolated from sediment collected at the continental shelf (depth = 322 m) off Wijdefjorden, Svalbard, during a marine-geoscientific cruise to North Spitsbergen in 2017. Cultivation in liquid media and the LC/MS-based chemical examination of the strain *Paenibacillus* sp. SVB7 identified the production of previously unreported molecules with the molecular ions at m/z 384. Scaling-up of the culture enabled us to purify two new compounds, svalbamides A and B, and subsequently elucidate their structures by spectroscopic analysis, chemical derivatization, and quantum mechanics-based calculation. Here, we report the structural determination of svalbamides A and B (**1**, **2**; Figure 1) along with their biological activity.

## 2. Results and Discussion

### 2.1. Phylogenetic Analysis

**Figure 2:**
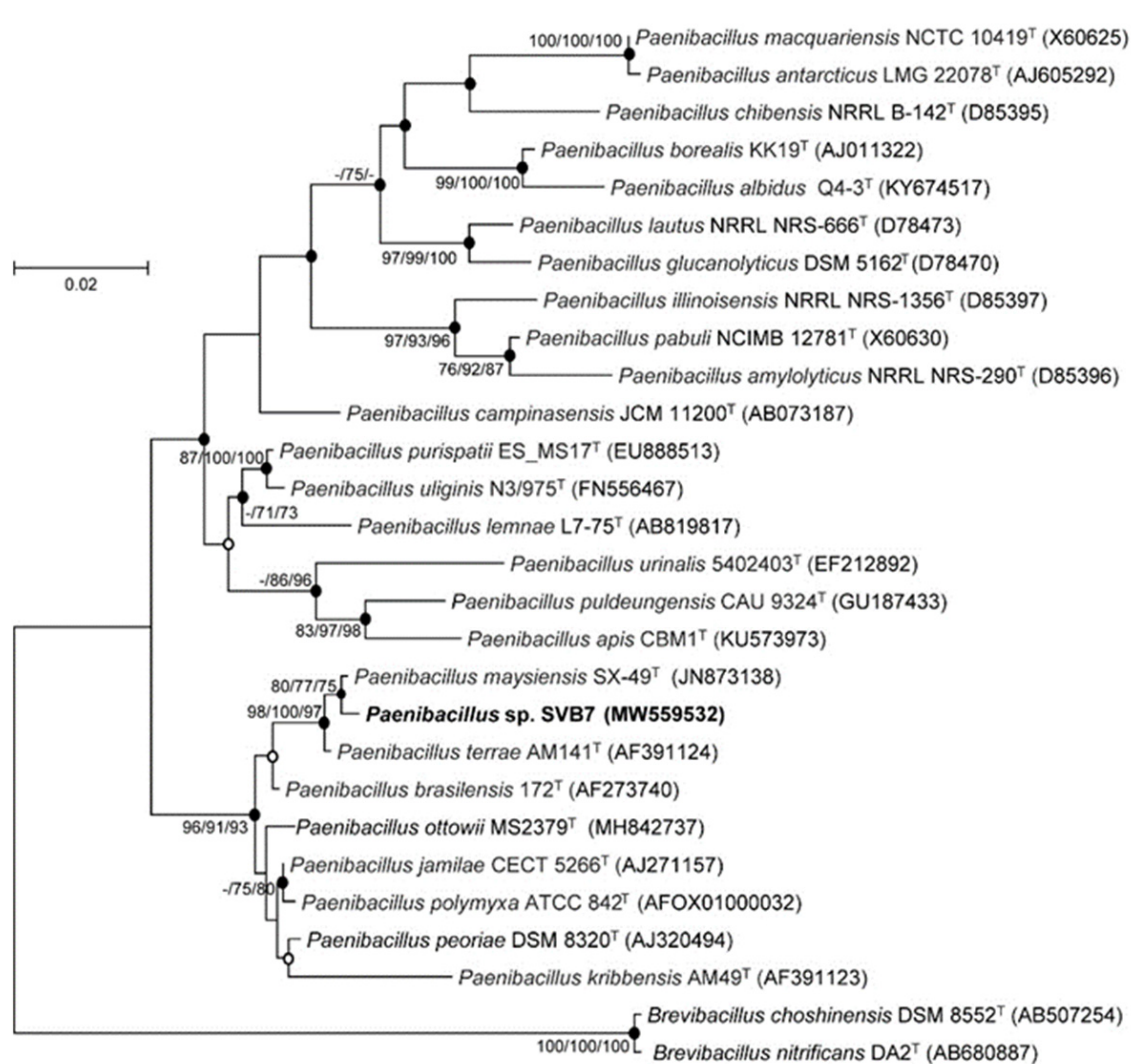
Maximum likelihood phylogenetic tree showing the position of *Paenibacillus* sp. SVB7. Bootstrap values (expressed as percentages of 1000 replications) over 70% are shown to the left of the node, and represent maximum likelihood, neighbor-joining, and minimum evolution (reading from left to right). Filled and open circles at each node indicate nodes recovered by all three treeing methods or by two treeing methods, respectively. Two 16S rRNA gene sequences of the genus *Brevibacillus* were used as outgroups. Bar, 0.02 substitutions per nucleotide position.

Sequence comparison using the almost complete 16S rRNA gene sequence of strain SVB7 (1440 bp) in BLASTn and EzBioCloud searches revealed that strain SVB7 belongs to the genus *Paenibacillus* of the Paenibacillaceae family. According to 16S rRNA gene sequence similarities, strain SVB7 was most closely related to *P. maysiensis* SX-49^T^ (99.30% similarity), followed by *P. terrae* AM141^T^ (98.26%), and *P. peoriae* DSM 8320^T^ (97.42%). In all phylogenetic trees inferred by maximum likelihood, neighbor-joining, and minimum-evolution methods, strain SVB7 was located within the *Paenibacillus* clade and formed a robust clade with *P. maysiensis* and *P. terrae*, providing clear support for its genus being classified as *Paenibacillus* (Figure 2). Based on the formation of the robust clade with *P. maysiensis* SX-49^T^ and > 98.7% 16S rRNA gene sequence similarity, it is likely that strain SVB7 is a member of *Paenibacillus maysiensis*. However, inclusion of strain SVB7 must be confirmed using whole-genome sequencing analysis.

### 2.2. Structural Elucidation

Svalbamide A (**1**) was isolated as a white powder. The molecular formula of **1** was assigned as C_20_H_37_N_3_O_4_, which has an unsaturation number of 4 based on high-resolution electrospray ionization (HR-ESI) mass spectrometry ([M + H]^+^ at *m*/*z* 384.2851, calculated as 384.2857) along with ^1^H and ^13^C NMR spectra. The ^13^C NMR spectra of **1** showed three carbonyl carbon (*δ*_C_ 174.2, 171.0, and 170.8), one oxygenated methine carbon (*δ*_C_ 67.5), and two α-amino methine carbon (*δ*_C_ 57.2 and 49.4) signals. Further analysis of these spectra revealed the existence of eight methylene carbon resonances (*δ*_C_ 43.4–25.2), two more methine carbons (*δ*_C_ 33.7 and 30.7), and four methyl carbons (*δ*_C_ 19.3, 19.1, 18.0, and 11.2) in the aliphatic region. The ^1^H and HSQC NMR spectra of **1** identified four exchangeable protons (*δ*_H_ 8.10, 7.83, 7.78, and 4.65), one carbinol proton (*δ*_H_ 3.78), two α-amino protons (*δ*_H_ 4.30 and 4.18), two more methine protons (*δ*_H_ 1.96 and 1.28), eight methylene protons (*δ*_H_ 1.05–3.16), and twelve methyl protons (*δ*_C_ 0.88, 0.84, 0.82, and 0.81). Based on the NMR spectroscopic features of the amide carbonyl carbons, α-amino groups, and many aliphatic signals, the structure of svalbamide A (**1**) was deduced as a peptide bearing an aliphatic chain.

**Figure 3:**
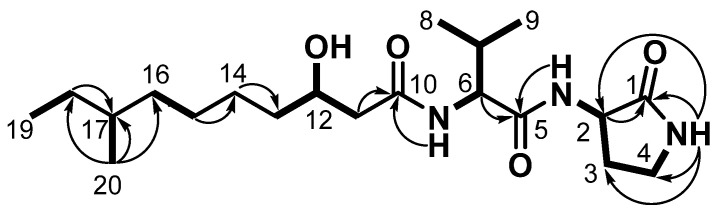
Key HMBC and COSY correlations of svalbamides A (**1**) and B (**2**).

The interpretation of COSY, TOCSY, and HMBC NMR spectra enabled us to determine the partial structures of **1**. First, the 2-NH (*δ*_H_ 8.10)/H-2 (*δ*_H_ 4.30) COSY correlation connected 2-NH to the C-2 α-carbon (*δ*_C_ 49.4). The TOCSY and COSY correlations among H-2, H-3a and H-3b (*δ*_H_ 1.82 and 2.26), H_2_-4 (*δ*_H_ 3.16), and 4-NH (*δ*_H_ 7.78) secured the spin system from 2-NH to 4-NH. The HMBC correlations from 4-NH to C-1 (*δ*_C_ 174.2), C-2 (*δ*_C_ 49.4), C-3 (*δ*_C_ 28.0), and C-4 (*δ*_C_ 38.0), and from 2-NH to C-1 (*δ*_C_ 174.2), led to elucidation of the substructure as a 3-amino-2-pyrrolidinone. The structure of valine was assigned based on the ^1^H–^1^H couplings of 6-NH (*δ*_H_ 7.83), H-6 (*δ*_H_ 4.18), H-7 (*δ*_H_ 1.96), H_3_-8 (*δ*_H_ 0.84), and H_3_-9 (*δ*_H_ 0.88) in the COSY and TOCSY NMR spectra along with the H-6/C-5 (*δ*_C_ 171.0) HMBC correlation. The remaining part of the molecule was composed of a lipophilic acyl chain. The protons in the substructure from C-11 to C-20 belong to a single spin system as revealed by COSY/TOCSY correlations of the protons. These protons showed correlation peaks with an exchangeable proton at *δ*_H_ 4.65 in the TOCSY spectrum, indicating that the exchangeable proton is also included in the spin system (Figure 3).

The COSY correlation of H-11a and H-11b (*δ*_H_ 2.23 and 2.29)/H-12 (*δ*_H_ 3.78), H-12/H_2_-13 (*δ*_H_ 1.33), and H-12/12-OH (*δ*_H_ 4.65) showed connectivity from C-11 to C-13, including 12-OH. The HMBC correlations of 12-OH (*δ*_H_ 4.65) to C-11 (*δ*_C_ 43.4), C-12 (*δ*_C_ 67.5), and C-13 (*δ*_C_ 36.7) confirmed the partial structure. C-11 was attached to the C-10 carbonyl carbon as inferred by the H-11a and H-11b/C-10 HMBC correlation. Due to the overlapping aliphatic signals from H_2_-13 to H-18a and H-18b, HMBC correlations played a pivotal role in identifying the planar structure of this linear section. The HMBC correlations from H-14a and H-14b (*δ*_H_ 1.24 and 1.34) to C-13 (*δ*_C_ 36.7), from H_2_-15 (*δ*_H_ 1.23) to C-14 (*δ*_C_ 25.2), and from H-16a and H-16b (*δ*_H_ 1.05 and 1.25) to C-15 (*δ*_C_ 26.5) revealed the connectivity from C-13 to C-16. In addition, the COSY ^1^H–^1^H couplings of H-17 (*δ*_H_ 1.28)/H_3_-20 (*δ*_H_ 0.81) and H-18a and H-18b (*δ*_H_ 1.09 and 1.28)/H_3_-19 (*δ*_H_ 0.82), along with the HMBC signals of H_3_-20 (*δ*_H_ 0.81) to C-16 (*δ*_C_ 36.0), C-17 (*δ*_C_ 33.7), and C-18 (*δ*_C_ 28.9), were finally assigned to 3-hydroxy-8-methyldecanoic acid (Figure 3). Consequently, the four unsaturation equivalents were fully explained by one pyrrolidinone ring containing one carbonyl group and two more carbonyl functional groups. Thus, svalbamide A (**1**) must not possess an additional ring and comprises a combination of the three substructures as a linear molecule.

Once the partial structures of 3-amino-2-pyrrolidinone, valine, and 3-hydroxy-8-methyldecanoic acid were identified, they were assembled according to the HMBC correlations: 2-NH (*δ*_H_ 8.10) of pyrrolidinone was correlated with the amide carbonyl carbon C-5 (*δ*_C_ 171.0) belonging to the valine residue, connecting 3-amino-2-pyrrolidinone to valine. The HMBC correlation from 6-NH (*δ*_H_ 7.83) of valine to the carbonyl carbon C-10 (*δ*_C_ 170.8) of 3-hydroxy-8-methyldecanoic acid established the sequence from valine to 3-hydroxy-8-methyldecanoic acid. Therefore, the planar structure of svalbamide A (**1**) was finally elucidated as a previously unreported lipodipeptide (Figure 3).

Svalbamide B (**2**) was isolated as a white powder, and its molecular formula was determined to be C_20_H_37_N_3_O_4_, which contains four double bond equivalents, using high-resolution electrospray ionization (HR-ESI) mass spectrometry ([M + H]^+^ at *m*/*z* 384.2845, calculated as 384.2857). This molecular formula was identical to that of svalbamide A (**1**). The ^1^H and ^13^C NMR data of **2** in DMSO-*d*_6_ were extremely similar to those of **1** (Table 1), but distinct differences in chemical shifts were found mainly in the 3-amino-2-pyrrolidinone unit. Specifically, H-2 in **1** (*δ*_H_ 4.30) was shifted upfield in **2** (*δ*_H_ 4.27), while signals for H-3a and H-3b in **1**, at *δ*_H_ 1.82 and 2.26, were detected at *δ*_H_ 1.76 and 2.29 in **2**. C-3 (*δ*_C_ 28.0) also shifted slightly to the deshielded region by 0.3 ppm in svalbamide B (**2**). Comprehensive analysis of 1D and 2D NMR data indicated the planar structure of **2** to be the same as **1** (Figure 3). Based on the observation that the distinct chemical shift differences were found in 3-amino-2-pyrrolidone unit, the structure of svalbamide B (**2**) was expected to have stereochemical modification in this residue.

The absolute configurations at the α-carbons of the two amino acid units were determined by applying the advanced Marfey’s method for derivatization with the l- and d- forms of 1-fluoro-2,4-dinitrophenyl-5-alanine amide (FDAA). LC/MS analysis of the FDAA derivatives of hydrolysates of **1** and **2** (Table S1) showed that they commonly possess d-valine. Because 3-amino-2-pyrrolidinone is converted into 2,4-diaminobutanoic acid during acid hydrolysis, an authentic sample of 2*S*,4-diaminobutanoic acid was derivatized with l- and d-FDAA to allow comparison. By comparing the retention times of the FDAA adducts of authentic 2*S*,4-diaminobutanoic acid, svalbamide A (**1**) was revealed to bear 3*R*-3-amino-2-pyrrolidinone, whereas svalbamide B (**2**) incorporates 3*S*-3-amino-2-pyrrolidinone (Figure 1).

**Figure 4:**
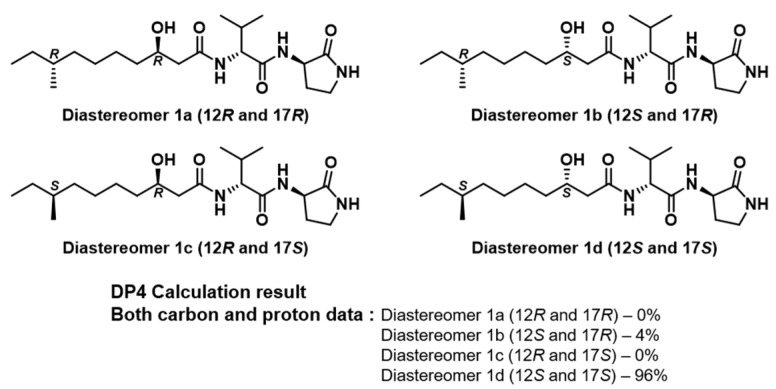
The simulated models of the four possible diastereomers (**a**–**d**) of svalbamide A **(1)** and the results of DP4 calculations.

The 3-hydroxy-8-methyldecanoic acid moiety contained stereogenic centers at C-12 and C-17. Initially, the modified Mosher’s method was applied for the oxygen-bearing chiral center at C-12. However, multiple esterifying attempts at the hydroxy group by *S*- and *R*-MTPA-Cl were not successful. Therefore, DP4 calculation was used to determine the absolute configurations. Four possible diastereomers of 3-hydroxy-8-methyldecanoic acid of svalbamide A (**1**), namely **1a** (12*R* and 17*R*), **1b** (12*S* and 17*R*), **1c** (12*R* and 17*S*), and **1d** (12*S* and 17*S*), were constructed with the established 2*R* and 6*R* configurations (Figure 4). Following this, the ^1^H and ^13^C chemical shifts of 158 conformers were calculated and averaged with their Boltzmann populations. Our DP4 calculations, based on statistical comparisons of the calculated and experimental chemical shifts, indicated that the diastereomer **1d** (12*S* and 17*S*) was suitable for svalbamide A (**1**) with 96.0% probability (Figure 4). The absolute configuration of svalbamide B (**2**) was subsequently proposed as 2*S*, 6*R*, 12*S,* and 17*S*.

### 2.3. Biological Evaluation

**Figure 5:**
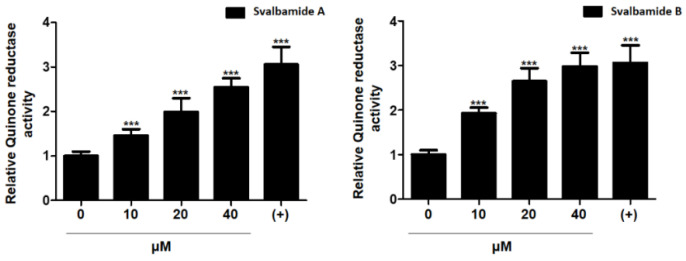
Induction of quinone reductase activity by svalbamide A (**1**) and B (**2**). Svalbamide A (**1**) and B (**2**) showed QR induction activity, with 2.54- and 2.98-fold increases, respectively, at 40 μM. All data represent the mean ± SD (*n* = 3). *** *p* < 0.001 compared to the control.

The biological activities of svalbamides A (**1**) and B (**2**) were evaluated in several ways. First, we measured cytotoxicity against various cancer cell lines [5], including HCT116 (human colorectal cancer cells), MDA-MB-231 (human breast cancer cells), A549 (human lung cancer cells), SK-HEP-1 (human liver cancer cells), and SNU-638 (human gastric cancer cells), but **1** and **2** showed no significant cytotoxicity against the tested cell lines even at 50 μM. Therefore, we evaluated the detoxification ability by measuring quinone reductase (QR) activity. QR is a major phase II detoxification enzyme, and the induction of QR activity is considered as a strategy to increase the chemoprevention effect. Svalbamide A (**1**) enhanced QR activity by 1.45-, 1.98-, and 2.54-fold at 10, 20, and 40 μM, respectively, in a concentration-dependent manner. In addition, svalbamide B (**2**) effectively induced QR activity by 1.93-, 2.64-, and 2.98-fold at 10, 20, and 40 μM, respectively (Figure 5). At a concentration of 40 μM, it exhibited a comparable level of QR activity induction in the positive control of 1 μM *β*-naphthoflavone (*β*-NF). These results suggest that **1** and **2** are potential chemotypes with chemopreventive activity.

## 3. Materials and Methods

### 3.1. General Experimental Procedures

Optical rotations were measured using a JASCO P-2000 polarimeter (sodium light source, JASCO, Easton, PA, USA) with a 1 cm cell. IR spectra were obtained using a Thermo NICOLET iS10 spectrometer (Thermo, Madison, CT, USA). ^1^H, ^13^C, and 2D NMR spectra were recorded on a Bruker Avance 800 MHz spectrometer (Bruker, Billerica, MA, USA) at the Research Institute of Pharmaceutical Sciences, Seoul National University. ESI low-resolution LC/MS data were recorded using an Agilent Technologies 6130 Quadrupole mass spectrometer (Agilent Technologies, Santa Clara, CA, USA) coupled with an Agilent Technologies 1200 series high-performance liquid chromatography (HPLC) instrument using a reversed-phase C_18_(2) column (Phenomenex Luna, 100 × 4.6 mm). HR-ESI mass spectra were acquired on a high-resolution LC/MS–MS spectrometer (Q-TOF 5600) at the National Instrumentation Center for Environmental Management (NICEM) in the College of Agriculture and Life Sciences at Seoul National University.

### 3.2. Isolation, Cultivation, Phylogenetic Analysis, and Extraction of Bacteria

**Figure 6:**
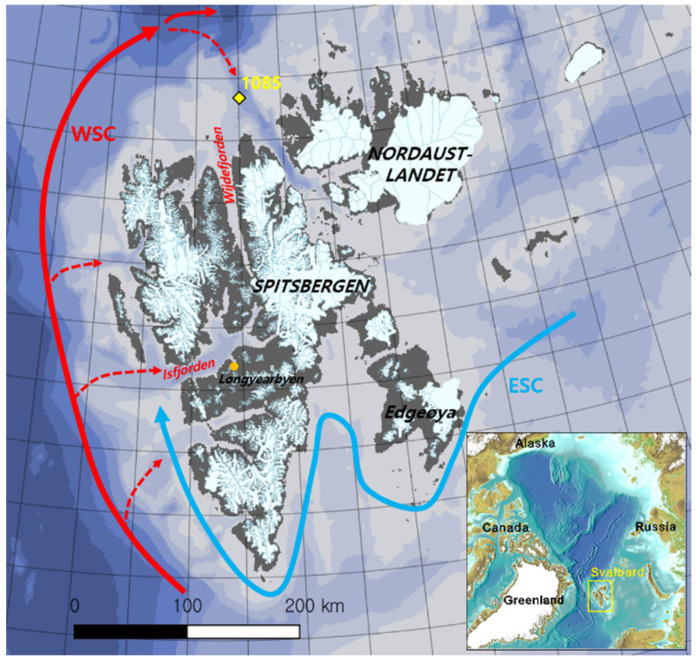
Map of the Svalbard archipelago with the core site and the main currents influencing Svalbard highlighted. The red and blue arrows indicate the West Spitsbergen Current (WSC) and East Spitsbergen Current (ESC), respectively, and the yellow rectangle indicates the coring site (HH17-1085-GC). The shaded white color represents the present glacier-covered areas on the archipelago.

During the Korea–Norway Joint marine-geoscientific cruise with RV Helmer Hanssen to North Spitsbergen in 2017, a sediment core (HH17-1085) was taken at a water depth of 322 m with a giant box corer from the continental shelf off Wijdefjorden (80°16.469’ N, 016°12.625’ E) in Svalbard (Figure 6). Surface sediment corresponding to 1 cm depth was collected from core HH17-1085. A portion of the sample (2 g) was diluted in 20 mL sterilized water and vortexed. The mixture was spread on YEME isolation solid medium (500 mL of sterilized water, 9 g agar, 100 mg cycloheximide, 2 g yeast, 5 g malt, and 2 g glucose) for two weeks. Strain SVB7 was isolated in YEME medium after one week of incubation for further study.

The 16S rRNA gene sequence of strain SVB7 was obtained by Sanger sequencing using PCR products amplified with the universal primers 27F and 1492R. The resultant 16S rRNA gene sequence (1440 bp) was queried in a BLASTn search implemented at GenBank and was also identified by the “16S-based ID service” in the EzbioCloud database [6]. For phylogenetic analysis, sequences of strain SVB7 and its close relatives retrieved from the EzBioCloud database were aligned with the SINA online aligner [7]. Using the aligned sequences, phylogenetic trees were inferred by maximum likelihood, neighbor-joining, and minimum-evolution algorithms implemented in MEGA software version 7.0 [8].

The SVB7 strain was cultured in 50 mL modified K medium (4 g yeast extract, 5 g malt extract, 5 g soytone, 5 g soluble starch, 5 g mannitol, 2 g glucose, and 6 g glycerol in 1 L deionized water) in a 125 mL Erlenmeyer flask. After cultivation for 2 days on a rotary shaker at 200 rpm and 30 °C, 5 mL of the culture medium was inoculated in 200 mL of modified K medium in a 500 mL Erlenmeyer flask. After cultivation for 2 days under the same incubation conditions, 10 mL of the culture medium was inoculated in 1 L of modified K medium in 2.8 L Fernbach flasks (200 ea × 1 L) at 170 rpm and 30 °C for 6 days. The whole culture of SVB7 was extracted with 300 L of EtOAc. The EtOAc and water layers were separated, and the remaining water in the EtOAc layer was removed by adding anhydrous sodium sulfate. The extract was concentrated using a rotary evaporator, yielding 50 g of dry material.

### 3.3. Isolation of Svalbamides A and B

The crude extract was divided into ten equal parts and fractioned over a C_18_ reversed-phase open column (*ϕ* 6.5 × 10 cm) with 500 mL of 20%, 40%, 60%, 80%, and 100% MeOH–H_2_O. The 80% MeOH fraction was subjected to a reversed-phase HPLC (Kromasil C_18_, 5 μm, 250 × 10 mm, flow rate = 2 mL/min) using a gradient solvent system from 35% to 75% CH_3_CN–H_2_O over 40 min with 210 nm UV detection. Svalbamides A and B were collected as one broad peak at 25 min. The recorded ^1^H NMR spectra of the peak indicated a diastereomeric mixture, prompting further purification on a chiral HPLC column (CHIRALPAK IB, 5 μm, 250 × 4.6 mm, flow rate = 0.6 mL/min) using a step gradient 45% CH_3_CN isocratic solvent system over 10 min, followed by a 60% CH_3_CN–H_2_O isocratic solvent system from 10 to 50 min. Svalbamides A (10.0 mg) and B (7.8 mg) were isolated at 30 and 31.5 min, respectively.

Svalbamide A (1): white powder; [α]20D +19.5 (c 0.1, MeOH); IR (neat) ν_max_ 3289, 2926,1632 cm^−1^; ^1^H and ^13^C NMR (800 MHz, DMSO-*d*_6_) (Table 1); HR-ESI–MS *m*/*z*: [M + H]^+^ Calcd for C_20_H_38_N_3_O_4_, 384.2857, found 384.2851.

Svalbamide B (2): white powder; [α]20D +25.2 (c 0.1, MeOH); IR (neat) ν_max_ 3276, 2928, 1612 cm^−1^; ^1^H and ^13^C NMR (800 MHz, DMSO-*d*_6_) (Table 1); HR-ESI–MS *m*/*z*: [M + H]^+^ Calcd for C_20_H_38_N_3_O_4_, 384.2857, found 384.2845.

### 3.4. Conformational Search and DP4 Analysis

A conformational search was carried out using a mixed sampling method of torsional/low-mode using MacroModel (version 9.9, Schrödinger LLC) in the Maestro suite (version 9.9, Schrödinger LLC). A total of 158 conformers of the diastereomers were identified with relative potential energies below 10 kJ/mol using the MMFF force field. The shielding tensor values of the optimized conformers were calculated based on the equation below, where $\delta_{calc}^{x}$ is the calculated NMR chemical shift for nucleus *x*, and *σ*^o^ is the shielding tensor for the proton and carbon nuclei calculated at the B3LYP/6-31++ level. These values were averaged via the Boltzmann population with the associated Gibbs free energy and utilized for the DP4 analysis, which was facilitated using an Excel spreadsheet provided by the authors of [9] and as described in their publication.

$$\delta_{calc}^{x}=\frac{\sigma {}^{\circ}-\sigma^{x}}{1-\sigma {}^{\circ}/10^{6}}$$

### 3.5. Quinone Reductase Assay

Hepa1c1c7 murine hepatoma cells (American Type Culture Collection, Manassas, VA, USA) were used to investigate QR induction activity. The test cells were seeded (3 × 10⁴ cells/mL) and incubated at 37 °C for 24 h with 5% CO_2_ containing humidified atmosphere. The plates were then exposed to svalbamides A and B (**1**, **2**), including a positive control compound, *β*-naphthoflavone (*β*-NF). After 24 h, the media were decanted from the wells, and the cells in each well were lysed by incubation at 37 °C with 250 μL of a mixed solution consisting of 10 mM Tris-HCl pH 8.0, 140 mM NaCl, 15 mM MgCl_2_, and 0.5% NP-40 (IGEPAL CA-630, Sigma, St. Louis, MO, USA) for 10 min. A 1 mL aliquot of the complete reaction mixture (12.5 mM Tris-HCl pH 7.4, 0.67 mg/mL bovine serum albumin (BSA), 0.01% Tween-20, 50 μM flavin adenine dinucleotide (FAD), 1 mM glucose-6-phosphate, 2 U/mL glucose-6-phosphate dehydrogenase, 30 μM NADP, 50 μg/mL 3-(4,5-dimethylthiazo-2-yl)-2,5-diphenyltetrazolium bromide (MTT), and 50 μM menadione) was added to each of the wells, and the plates were incubated at 25 °C for the colorimetric reaction. The rate of NADPH-dependent menadiol-mediated reduction of MTT in this reaction was measured at 610 nm, and cytotoxicity was determined by crystal violet staining of an identical set of the test plates. The quinone reductase activity was calculated from the following equation: absorbance change for MTT per min/absorbance of crystal violet × 3345 nmol/mg. The value of 3345 nmol/mg represents the ratio of the extinction coefficient of MTT and the proportionality constant of crystal violet. The relative QR activity was normalized using controls [10].

## 4. Conclusions

Our chemical study of the Arctic sediment-derived *Paenibacillus* sp. SVB7 led to the discovery and structural elucidation of two new pyrrolidinone-bearing lipodipeptides, svalbamides A (**1**) and B (**2**), in which QR activity could be induced. Based on spectroscopic analysis, advanced Marfey’s analysis, and DP4 calculation, these two compounds were identified to have a diastereomeric relationship with alternative absolute configurations at the 3-amino-2-pyrrolidione unit. Svalbamides A and B are structurally unique as they contain 3-amino-2- pyrrolidinone amino acid. This amino acid unit was rarely reported in natural products, with the only example being of actinoramide E, an antimalarial peptide from a marine-derived *Streptomyces* strain [11]. 3-Hydroxy-8-methyldecanoic acid is another interesting component. This saturated fatty acid was occasionally found in natural products from *Paenibacillus* and related bacteria. For example, tridecaptins A–C were first isolated from *Bacillus polymyxa* in 1978 and were studied in various fields, with reports of new derivatives and biosynthesis undertaken to date [12]. A series of new tridecaptin compounds containing 3-hydroxy-8-methyldecanoic acid were discovered in a *Paenibacillus* strain collected in the deep oligotrophic Krubera-Voronja cave. However, the absolute configuration of this fatty acid was not determined [13,14,15]. Octapeptin and cerexin from the *Bacillus* sp. bear the same fatty acid, but no experiments have yet been conducted to reveal the absolute stereochemistry [16,17]. Therefore, svalbamides A and B are the first metabolites for which the stereochemistry of 3-hydroxy-8-methyldecanoic acid was addressed. Discovering these new bioactive secondary metabolites from *Paenibacillus* from the polar region indicates that chemical studies of underinvestigated bacterial taxa in marine extreme habitats, such as the Arctic Ocean, could lead to the discovery of significant natural chemical diversity with pharmaceutical potential in terms of drug discovery.

## Figures and Tables

**Table 1:** ^1^H and ^13^C NMR data for svalbamides A (**1**) and B (**2**) in DMSO-*d*_6_.

			Svalbamide A (1) ^a^		Svalbamide B (2) ^a^	
Position			*δ*_C_, Type	*δ*_H,_ Mult (*J* in Hz)	*δ*_C_, Type	*δ*_H,_ Mult (*J* in Hz)
3-amino-2-pyrrolidinone		1	174.2, C		174.2, C	
		2	49.4, CH	4.30, m	49.5, CH	4.27, m
		3a	28.0, CH_2_	1.82, m	28.3, CH_2_	1.76, m
		3b		2.26, m		2.29, m
		4	38.0, CH_2_	3.16, m	38.0, CH_2_	3.16, m
		4-NH		7.78, br s		7.81, br s
		2-NH		8.10, d (8.5)		8.21, d (8.5)
d-Val		5	171.0, C		171.0, C	
		6	57.2, CH	4.18, dd (9.0, 6.5)	57.2, CH	4.20, dd (9.0, 6.5)
		7	30.7, CH	1.96, m	30.6, CH	1.94, m
		8	18.0, CH_3_	0.84, d (7.0)	18.0, CH_3_	0.83, d (7.0)
		9	19.3, CH_3_	0.88, d (7.0)	19.1, CH_3_	0.84, d (7.0)
		6-NH		7.83, d (9.0)		7.85, d (9.0)
3-hydroxy-8-methyldecanoic acid		10	170.8, C		170.8, C	
		11a	43.4, CH_2_	2.23, dd (14.0, 7.0)	43.4, CH_2_	2.25, dd (14.0, 7.0)
		11b		2.29, dd (14.0, 5.0)		2.28 dd (14.0, 5.0)
		12	67.5, CH	3.78, m	67.6, CH	3.78, m
		13	36.7, CH_2_	1.33, m ^b^	36.7, CH_2_	1.33, m ^b^
		14a	25.2, CH_2_	1.24, m ^b^	25.2, CH_2_	1.24, m ^b^
		14b		1.34, m ^b^		1.34, m ^b^
		15	26.5, CH_2_	1.23, m ^b^	26.5, CH_2_	1.23, m ^b^
		16a	36.0, CH_2_	1.05, m	36.0, CH_2_	1.05, m
		16b		1.25, m ^b^		1.25, m ^b^
		17	33.7, CH	1.28, m ^b^	33.7, CH	1.28, m ^b^
		18a	28.9, CH_2_	1.09, m	28.9, CH_2_	1.09, m
		18b		1.28, m ^b^		1.28, m ^b^
		19	11.2, CH_3_	0.82, t (7.0)	11.2, CH_3_	0.83, t (7.0)
		20	19.1, CH_3_	0.81, d (6.5)	19.1, CH_3_	0.81, d (6.5)
		12-OH		4.65, d (5.0)		4.67, d (5.0)

^a 1^H and ^13^C NMR data were recorded at 800 and 200 MHz, respectively. ^b^ Overlapping signals.

## Data Availability

All data is contained within this article and Supplementary Materials.

## Supplementary Materials

The following are available online at https://www.mdpi.com/article/10.3390/md19040229/s1, Figure S1. ^1^H NMR spectrum (800 MHz) of svalbamide A (**1**) in DMSO-*d*_6_., Table S1: LC/MS analysis of d- and l-FDAA derivatives of the amino acid-derived units in svalbamide A (**1**), svalbamide B (**2**), l-2,4-diamino butanoic acid (**3**), d-valine (**4**) and l-valine (**5**) authentic samples. Retention times (min) are notified., Figure S2. ^13^C NMR spectrum (200 MHz) of svalbamide A (**1**) in DMSO-*d*_6_, Figure S3. COSY NMR spectrum (800 MHz) of svalbamide A (**1**) in DMSO-*d*_6_, Figure S4. HSQC NMR spectrum (800 MHz) of svalbamide A (**1**) in DMSO-*d*_6_, Figure S5. HMBC NMR spectrum (800 MHz) of svalbamide A (**1**) in DMSO-*d*_6_, Figure S6. TOCSY NMR spectrum (800 MHz) of svalbamide A (**1**) in DMSO-*d*_6_, Figure S7. ^1^H NMR spectrum (800 MHz) of svalbamide B (**2**) in DMSO-*d*_6_, Figure S8. ^13^C NMR spectrum (200 MHz) of svalbamide B (**2**) in DMSO-*d*_6_, Figure S9. COSY NMR spectrum (800 MHz) of svalbamide B (**2**) in DMSO-*d*_6_, Figure S10. HSQC NMR spectrum (800 MHz) of svalbamide B (**2**) in DMSO-*d*_6_, Figure S11. HMBC NMR spectrum (800 MHz) of svalbamide B (**2**) in DMSO-*d*_6_, Figure S12. TOCSY NMR spectrum (800 MHz) of svalbamide B (**2**) in DMSO-*d*_6_, Figure S13. The simulated models of four possible diastereomers (**a**–**d**) of svalbamide A (**1**) and the result of DP4 calculation, Table S1. LC/MS analysis of d- and l-FDAA derivatives of the amino acid-derived units in svalbamide A (**1**), svalbamide B (**2**) and l-2,4-diamino butanoic acid authentic sample (**3**), d-valine (**4**) and l-valine (**5**). Retention times (min) are notified, Table S2. The major conformers of diastereomers (**a**–**d**) of svalbamide A (**1**), identified by conformational searches in MMFF94 force field using MacroModel_,_ Table S3. Experimental (Exp.) and calculated (Cal.) chemical shift values (CS, *δ*) of diastereomers (**a**–**d**) of **1** and svalbamide A (**1**).
