# Supplementary material for: Svalbamides A and B, Pyrrolidinone-Bearing Lipodipeptides from Arctic Paenibacillus sp
Source: Mar Drugs. 2021 Apr 17;19(4):229. doi: 10.3390/md19040229 (PMC8073366; doi:10.3390/md19040229)
Supplement: Supplementary file 1 [file marinedrugs-19-00229-s001.zip › marinedrugs-1183549-supplementary.pdf]

Supplementary Material

Svalbamides A and B, Pyrrolidinone-Bearing Lipodipeptides from Arctic *Paenibacillus* sp.

Young Eun Du[1], Eun Seo Bae[1], Yeonjung Lim[2], Jang-Cheon Cho[2], Sang-Jip Nam[3], Jongheon Shin[1], Sang Kook Lee[1], Seung-Il Nam[4] and Dong-Chan Oh[1,*]

[1] Natural Products Research Institute, College of Pharmacy, Seoul National University, Seoul 08826, Republic of Korea

[2] Department of Biological Sciences, Inha University, Incheon 22212, Republic of Korea

[3] Department of Chemistry and Nanoscience Ewha Womans University, Seoul 03760, Republic of Korea

[4] Korea Polar Research Institute, Incheon 21990, Republic of Korea

Table of Contents

Figure S1. ^{1}H NMR spectrum (800 MHz) of svalbamide A (**1**) in DMSO-d_6. ……… **S3**

Figure S2. ^{13}C NMR spectrum (200 MHz) of svalbamide A (**1**) in DMSO-d_6. ……. **S4**

Figure S3. COSY NMR spectrum (800 MHz) of svalbamide A (**1**) in DMSO-d_6. … **S5**

Figure S4. HSQC NMR spectrum (800 MHz) of svalbamide A (**1**) in DMSO-d_6. … **S5**

Figure S5. HMBC NMR spectrum (800 MHz) of svalbamide A (**1**) in DMSO-d_6. … **S6**

Figure S6. TOCSY NMR spectrum (800 MHz) of svalbamide A (**1**) in DMSO-d_6. . **S6**

Figure S7. ^{1}H NMR spectrum (800 MHz) of svalbamide B (**2**) in DMSO-d_6. ……… **S7**

Figure S8. ^{13}C NMR spectrum (200 MHz) of svalbamide B (**2**) in DMSO-d_6. …… **S8**

Figure S9. COSY NMR spectrum (800 MHz) of svalbamide B (**2**) in DMSO-d_6. ... **S9**

Figure S10. HSQC NMR spectrum (800 MHz) of svalbamide B (**2**) in DMSO-d_6. . **S9**

Figure S11. HMBC NMR spectrum (800 MHz) of svalbamide B (**2**) in DMSO-d_6. ... **S10**

Figure S12. TOCSY NMR spectrum (800 MHz) of svalbamide B (**2**) in DMSO-d_6. **S10**

Table S1. LC/MS analysis of D- and L-FDAA derivatives of the amino acid-derived units in svalbamide A (**1**), svalbamide B (**2**), L-2,4-diamino butanoic acid (**3**), D-valine (**4**), and L-valine (**5**) authentic samples. Retention times (min) are notified.……………………………………………………………………….. **S11**

Figure S13. The simulated models of four possible diastereomers (**a**–**d**) of svalbamide A (**1**) and the result of DP4 calculation. ……………………………….. **S12**

Table S2. The major conformers of diastereomers (**a**–**d**) of svalbamide A (**1**), identified by conformational searches in MMFF94 force field using MacroModel. ... **S13**

Table S3. Experimental (Exp.) and calculated (Cal.) chemical shift values (CS, δ) of diastereomers (**a–d**) of **1** and svalbamide A (**1**). ………………………………….. **S17**

Figure S1. ^{1}H NMR spectrum (800 MHz) of svalbamide A (**1**) in DMSO-d_6.

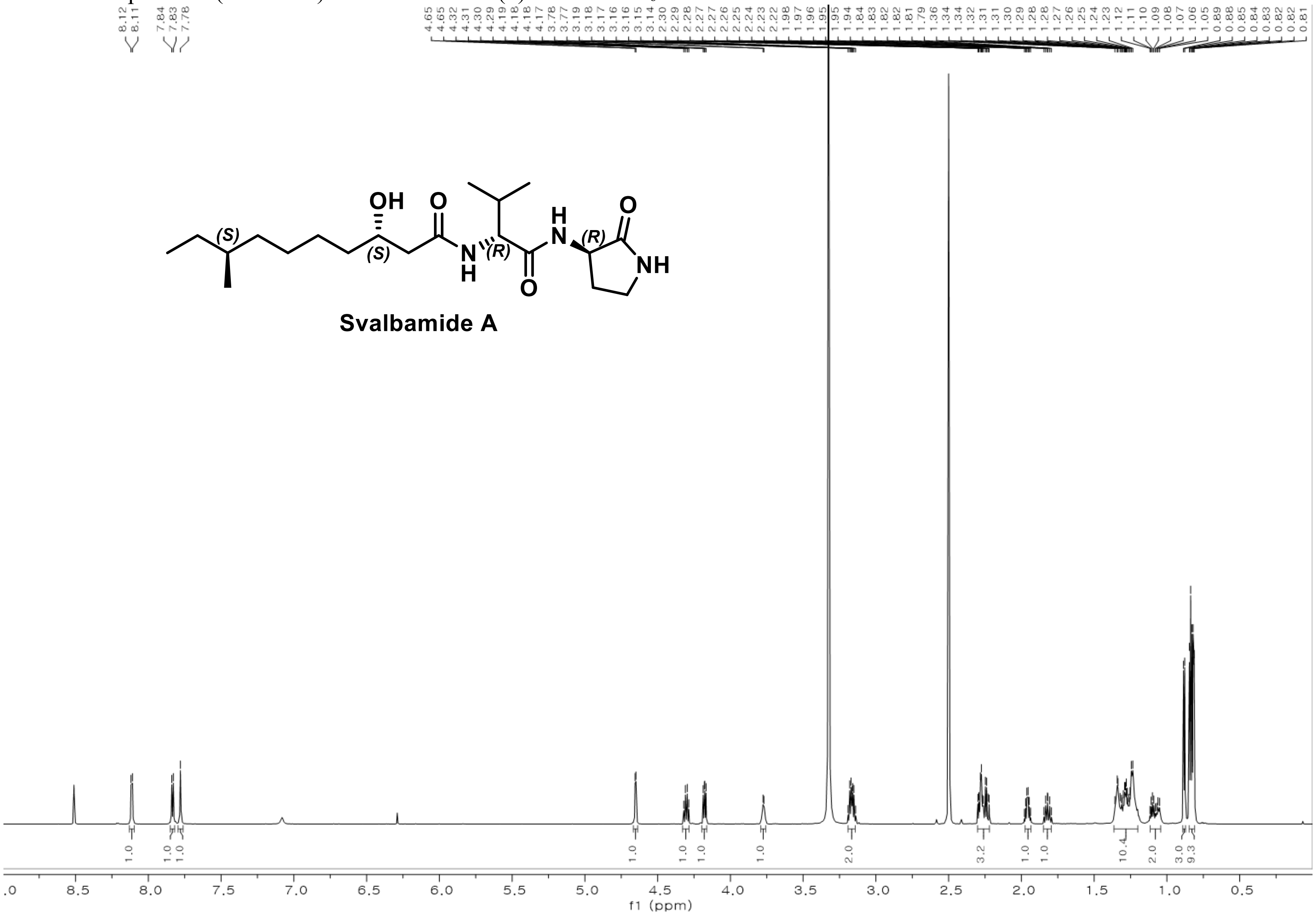

Figure S2. ^{13}C NMR spectrum (200 MHz) of svalbamide A (**1**) in DMSO-d_6.

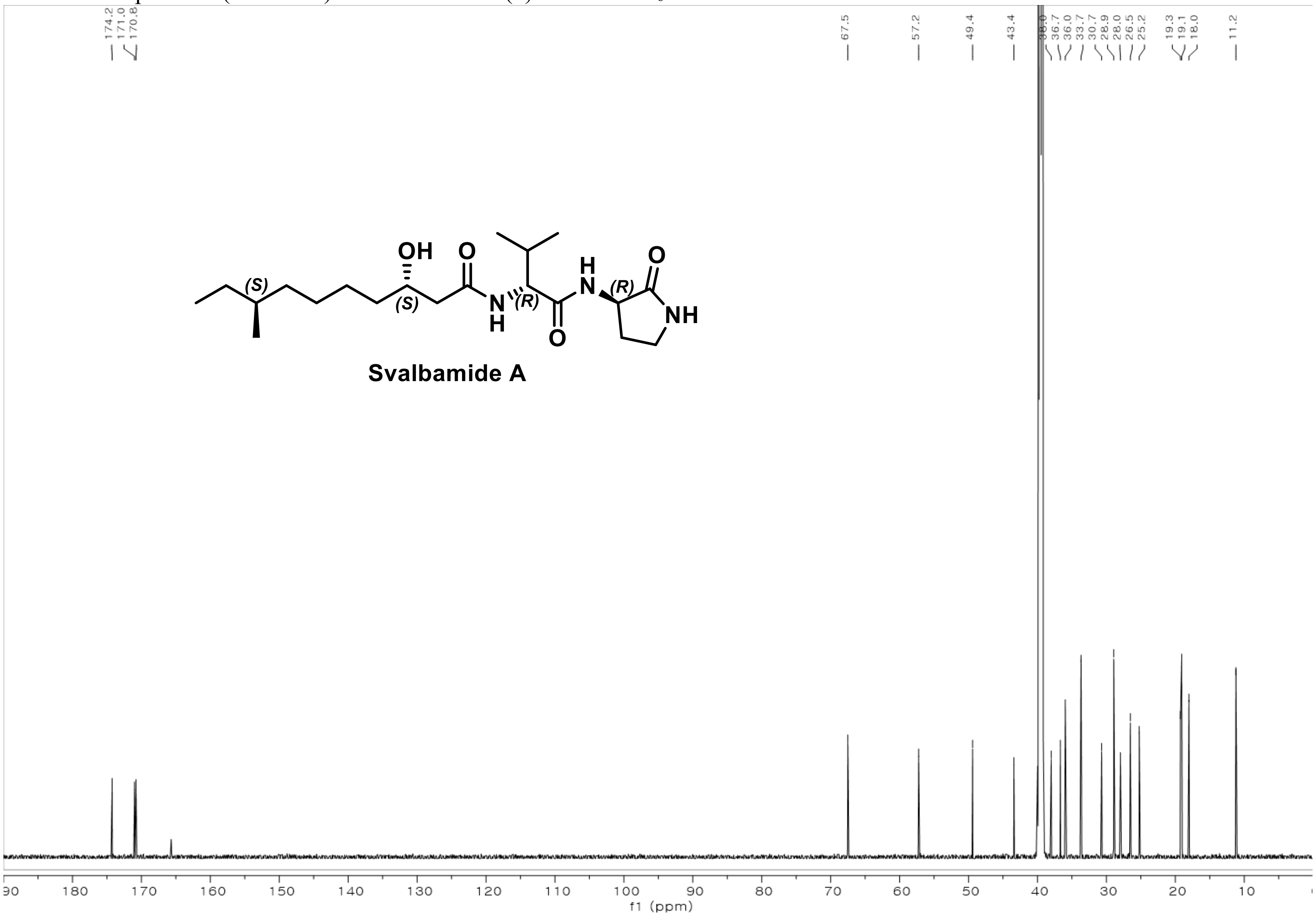

Figure S3. COSY NMR spectrum (800 MHz) of svalbamide A (**1**) in DMSO-d_6.

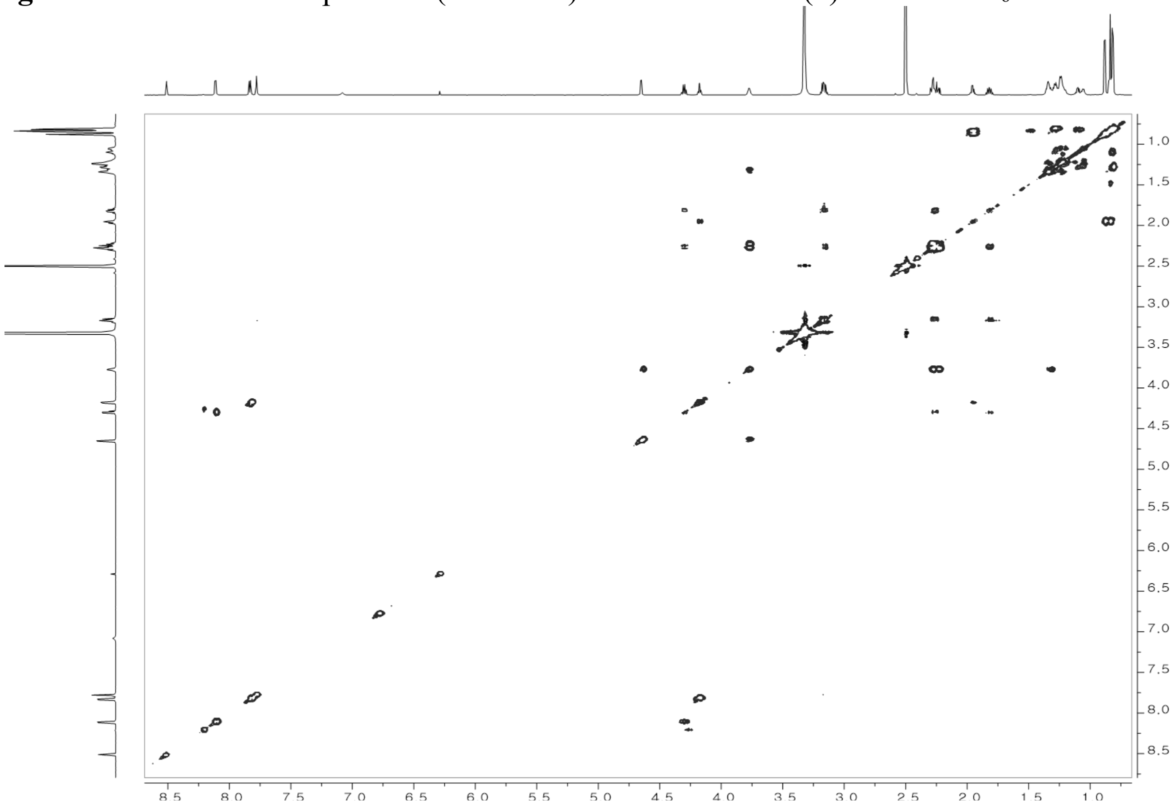

Figure S4. HSQC NMR spectrum (800 MHz) of svalbamide A (**1**) in DMSO-d_6.

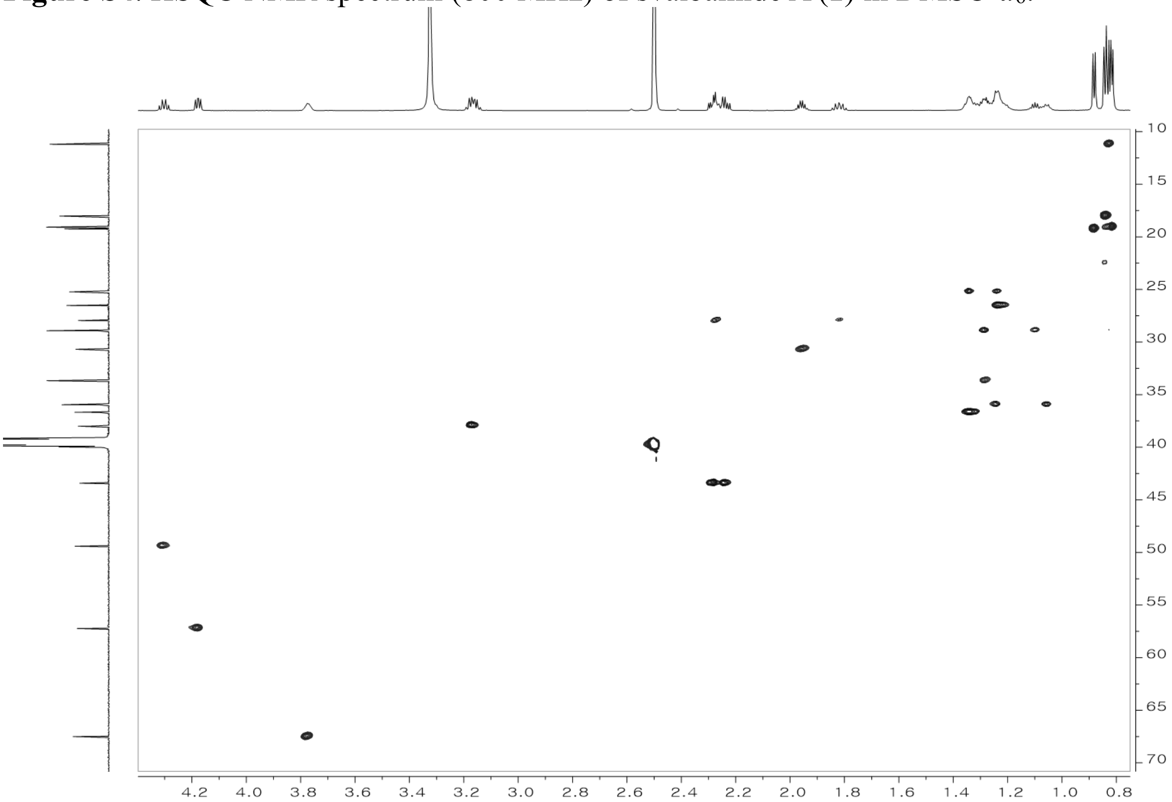

Figure S5. HMBC NMR spectrum (800 MHz) of svalbamide A (**1**) in DMSO-d_6.

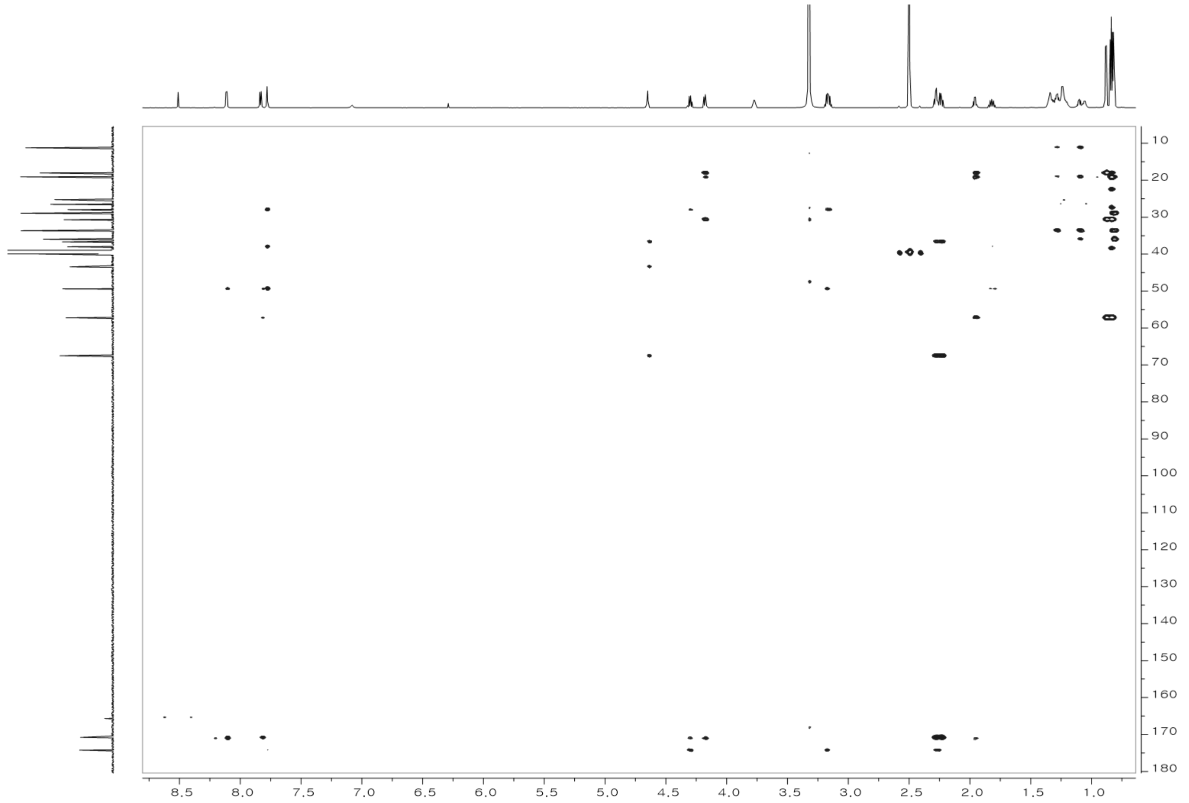

Figure S6. TOCSY NMR spectrum (800 MHz) of svalbamide A (**1**) in DMSO-d_6.

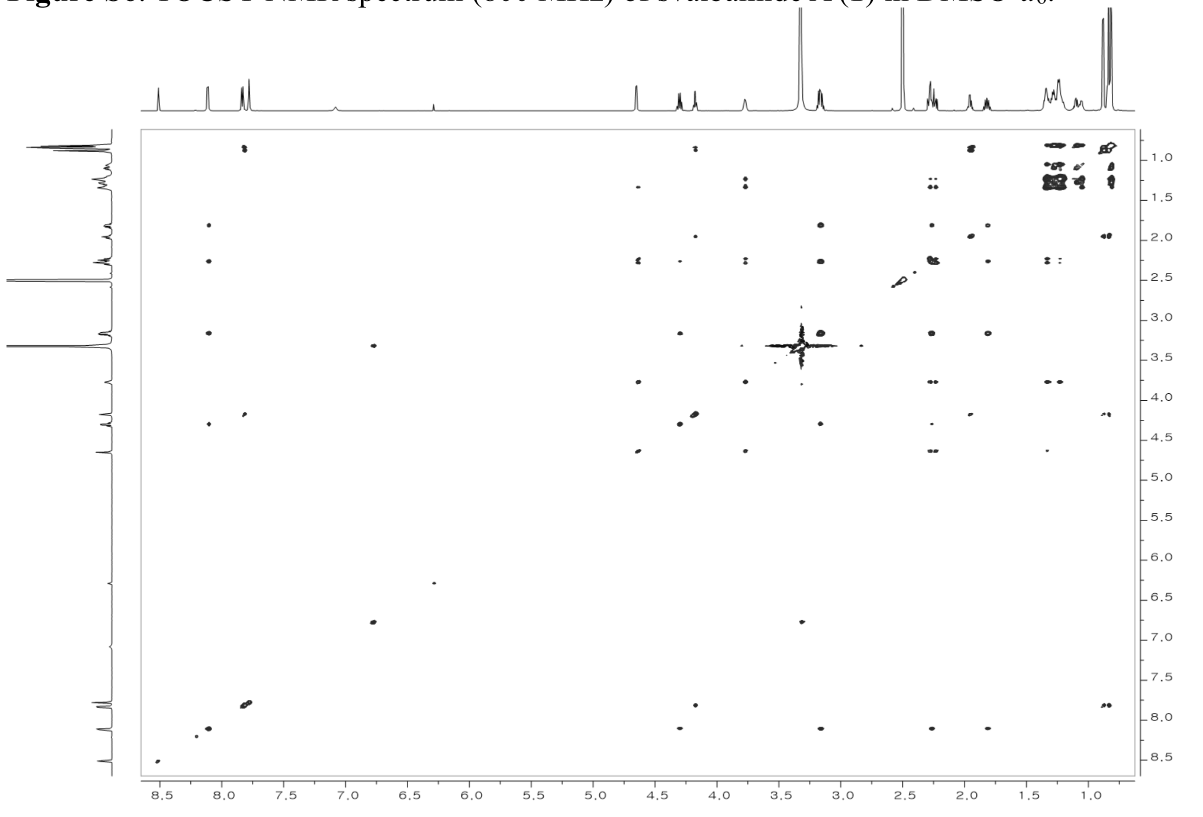

Figure S7. ^{1}H NMR spectrum (800 MHz) of svalbamide B (**2**) in DMSO-d_6.

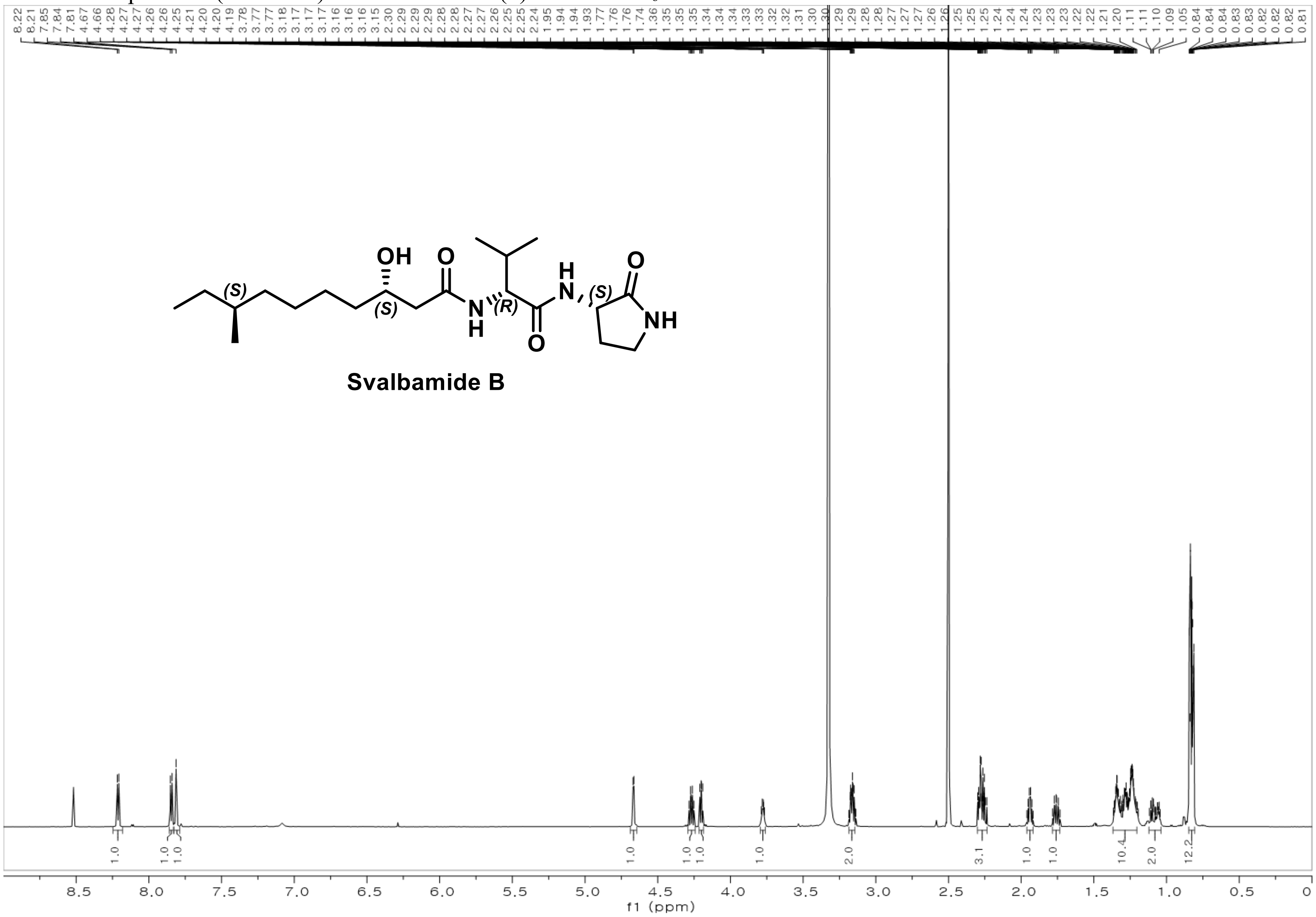

Figure S8. ^{13}C NMR spectrum (200 MHz) of svalbamide B (**2**) in DMSO-d_6.

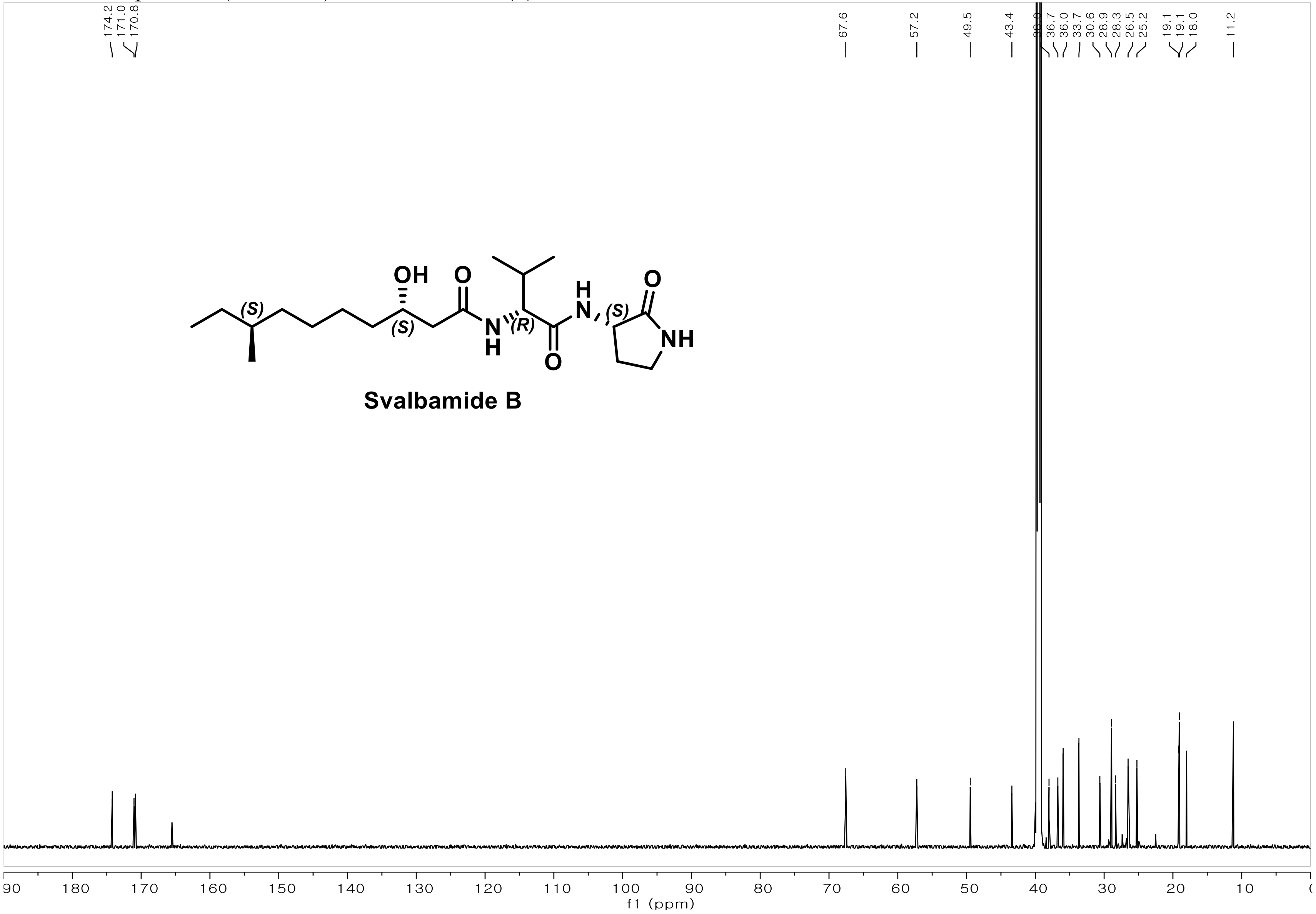

Figure S9. COSY NMR spectrum (800 MHz) of svalbamide B (**2**) in DMSO-d_6.

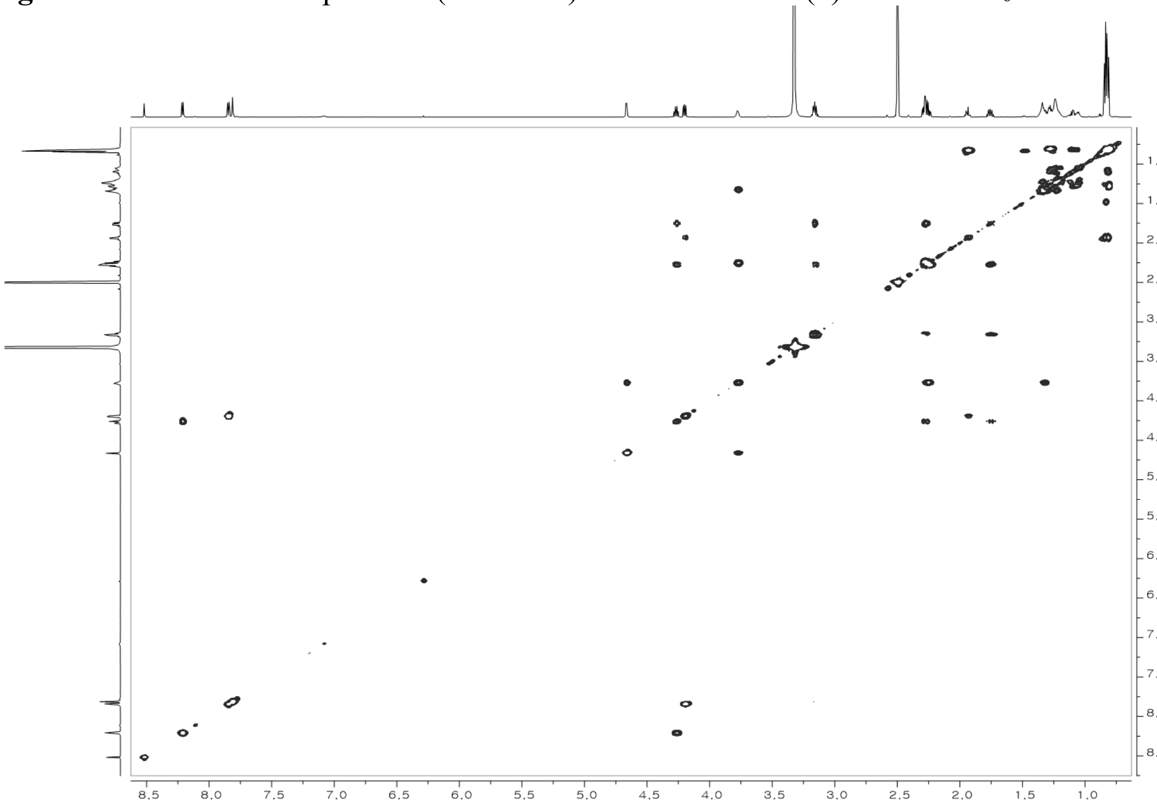

Figure S10. HSQC NMR spectrum (800 MHz) of svalbamide B (**2**) in DMSO-d_6.

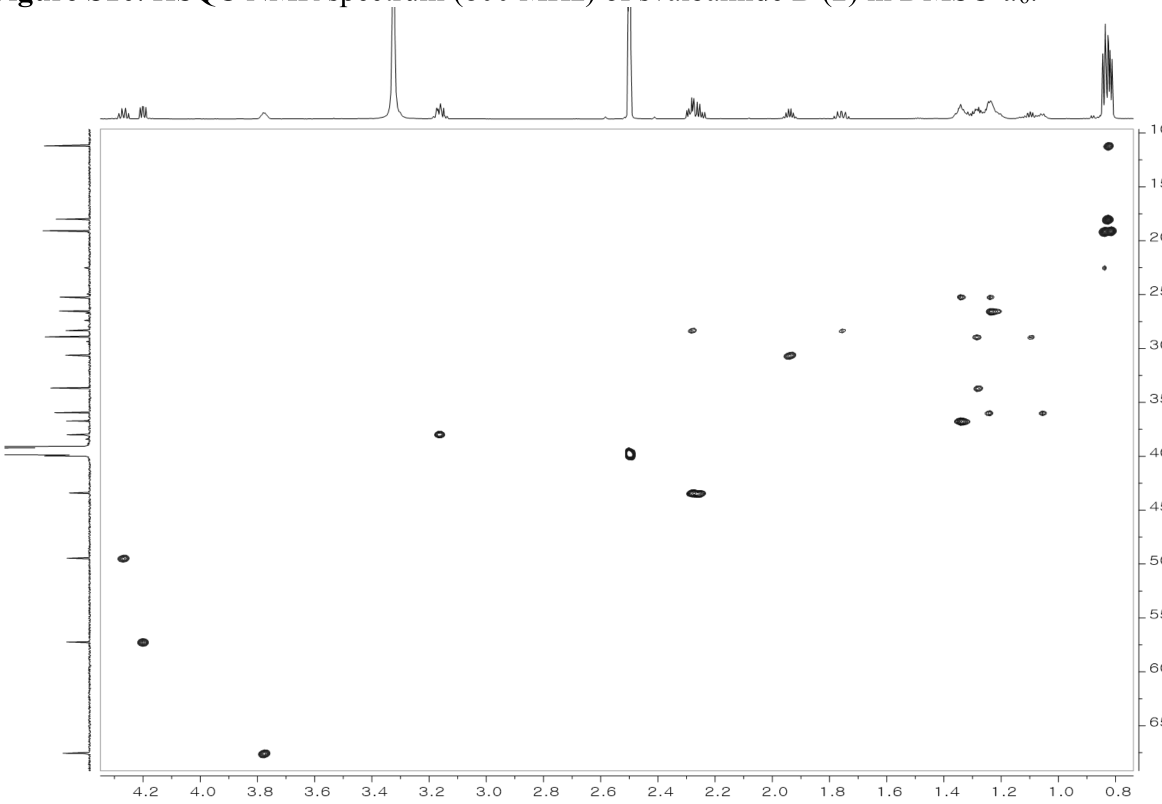

Figure S11. HMBC NMR spectrum (800 MHz) of svalbamide B (**2**) in DMSO-d_6.

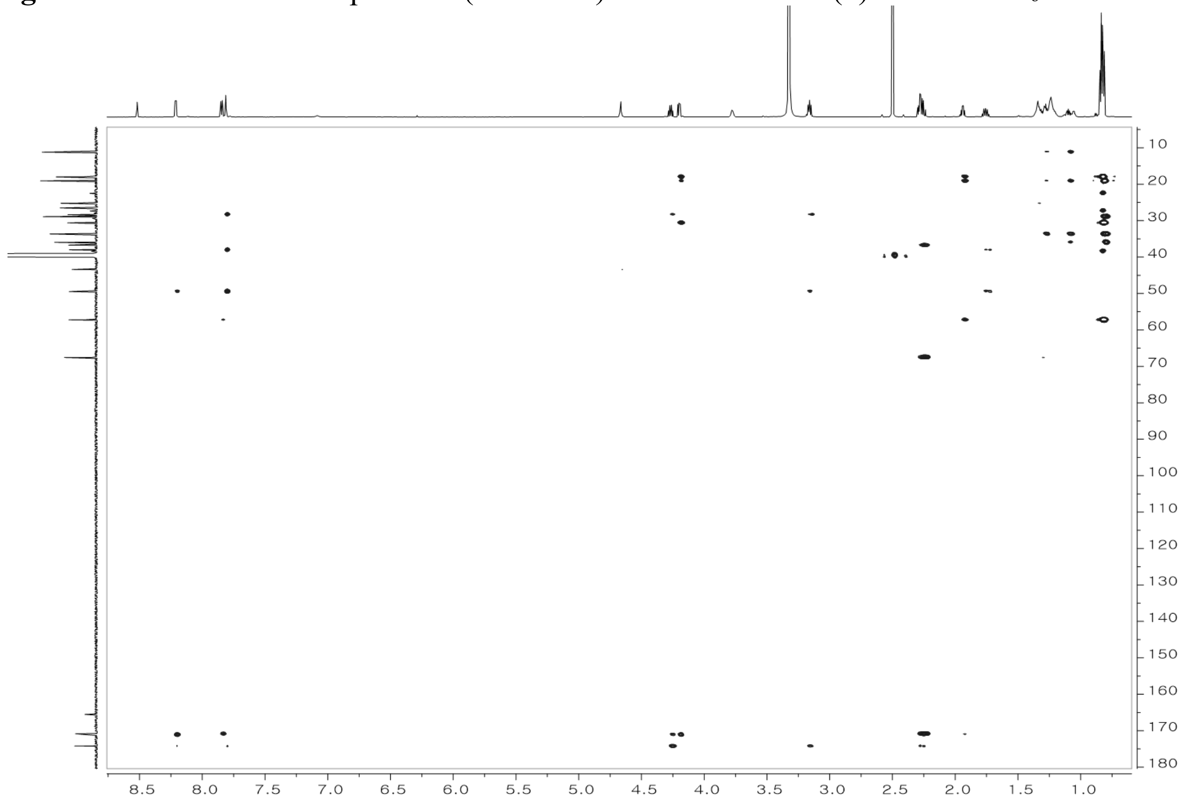

Figure S12. TOCSY NMR spectrum (800 MHz) of svalbamide B (**2**) in DMSO-d_6.

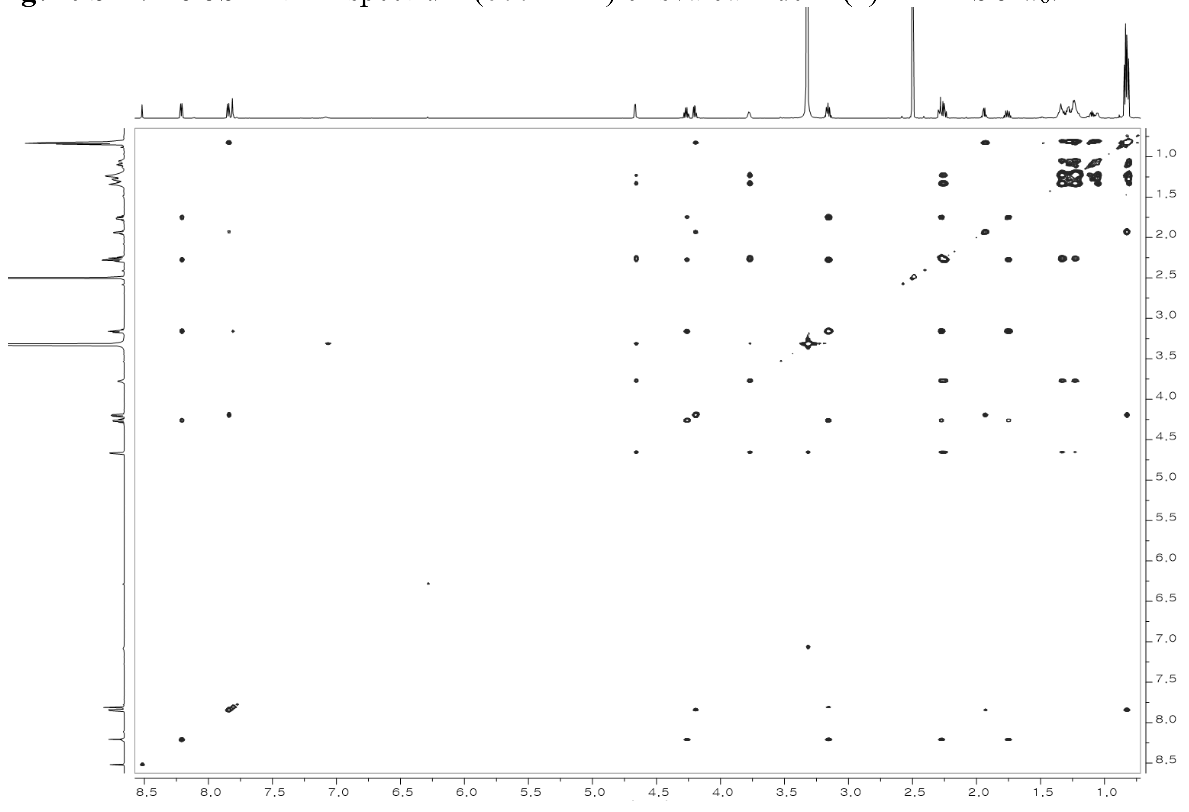

Table S1. LC/MS analysis of D- and L-FDAA derivatives of the amino acid-derived units in svalbamide A (**1**), svalbamide B (**2**), L-2,4-diamino butanoic acid (**3**), D-valine (**4**), and L-valine (**5**) authentic samples. Retention times (min) are notified.

Svalbamide A (**1**)	Valine	2,4-diamino butanoic acid
+ D-FDAA	29.2	33.1
+ L-FDAA	33.5	34.8
Elution order	D → L	D → L
Svalbamide (**2**)	Valine	2,4-diamino butanoic acid
+ D-FDAA	29.7	35.2
+ L-FDAA	33.0	33.1
Elution order	D → L	L → D

	L-2,4-diamino butanoic acid authentic sample (**3**)
+ D-FDAA	35.3
+ L-FDAA	33.6
Elution order	L → D

	D-Valine authentic sample (**4**)	L-Valine authentic sample (**5**)
+ D-FDAA	28.9	33.0
+ L-FDAA	33.0	28.9
Elution order	D → L	L → D

Figure S13. The simulated models of four possible diastereomers (**a**–**d**) of svalbamide A (**1**) and the result of DP4 calculation.

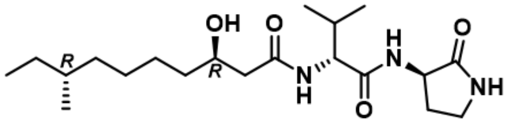

Diastereomer 1a (12*R* and 17*R*)

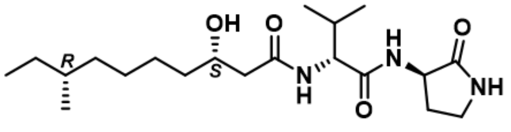

Diastereomer 1b (12*S* and 17*R*)

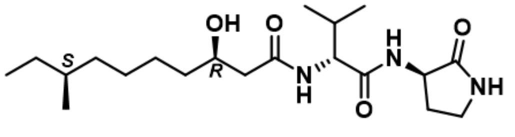

Diastereomer 1c (12*R* and 17*S*)

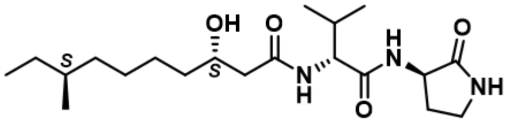

Diastereomer 1d (12*S* and 17*S*)

DP4 Calculation result

Both carbon and proton data : Diastereomer 1a (12*R* and 17*R*) – 0%
Diastereomer 1b (12*S* and 17*R*) – 4%
Diastereomer 1c (12*R* and 17*S*) – 0%
Diastereomer 1d (12*S* and 17*S*) – 96%

Carbon only data :

Diastereomer 1a (12*R* and 17*R*) – 0%
Diastereomer 1b (12*S* and 17*R*) – 57.9%
Diastereomer 1c (12*R* and 17*S*) – 1.7%
Diastereomer 1d (12*S* and 17*S*) – 40.3%

Proton only data :

Diastereomer 1a (12*R* and 17*R*) – 0%
Diastereomer 1b (12*S* and 17*R*) – 2.8%
Diastereomer 1c (12*R* and 17*S*) – 0%
Diastereomer 1d (12*S* and 17*S*) – 97.2%

Table S2. The major conformers of diastereomers (**a**–**d**) of svalbamide A (**1**), identified by conformational searches in MMFF94 force field using MacroModel.

Conformers[a]	Boltzmann population (%)[b]
Diastereomer 1a_1	22.964
Diastereomer 1a_2	16.8
Diastereomer 1a_3	15.144
Diastereomer 1a_4	12.335
Diastereomer 1a_5	7.154
Diastereomer 1a_6	5.711
Diastereomer 1a_7	4.117
Diastereomer 1a_8	3.754
Diastereomer 1a_9	3.301
Diastereomer 1a_10	2.451
Diastereomer 1a_11	1.605
Diastereomer 1a_12	1.499
Diastereomer 1a_13	0.981
Diastereomer 1a_14	0.648
Diastereomer 1a_15	0.57
Diastereomer 1a_16	0.542
Diastereomer 1a_17	0.424
Diastereomer 1b_1	14.011
Diastereomer 1b_2	7.726
Diastereomer 1b_3	6.735
Diastereomer 1b_4	6.003
Diastereomer 1b_5	5.089
Diastereomer 1b_6	5.089
Diastereomer 1b_7	4.988
Diastereomer 1b_8	4.697
Diastereomer 1b_9	2.963
Diastereomer 1b_10	2.918
Diastereomer 1b_11	2.898
Diastereomer 1b_12	2.489
Diastereomer 1b_13	2.407
Diastereomer 1b_14	2.395
Diastereomer 1b_15	2.23
Diastereomer 1b_16	2.148
Diastereomer 1b_17	1.875
Diastereomer 1b_18	1.873
Diastereomer 1b_19	1.87
Diastereomer 1b_20	1.777
Diastereomer 1b_21	1.617
Diastereomer 1b_22	1.351
Diastereomer 1b_23	1.186
Diastereomer 1b_24	1.167
Diastereomer 1b_25	1.164

Diastereomer 1b_26	1.131
Diastereomer 1b_27	1.044
Diastereomer 1b_28	0.963
Diastereomer 1b_29	0.86
Diastereomer 1b_30	0.856
Diastereomer 1b_31	0.658
Diastereomer 1b_32	0.541
Diastereomer 1b_33	0.529
Diastereomer 1b_34	0.526
Diastereomer 1b_35	0.416
Diastereomer 1b_36	0.39
Diastereomer 1b_37	0.387
Diastereomer 1b_38	0.365
Diastereomer 1b_39	0.35
Diastereomer 1b_40	0.35
Diastereomer 1b_41	0.313
Diastereomer 1b_42	0.3
Diastereomer 1b_43	0.289
Diastereomer 1b_44	0.276
Diastereomer 1b_45	0.272
Diastereomer 1b_46	0.271
Diastereomer 1b_47	0.249
Diastereomer 1c_1	39.844
Diastereomer 1c_2	23.166
Diastereomer 1c_3	3.189
Diastereomer 1c_4	3.049
Diastereomer 1c_5	2.886
Diastereomer 1c_6	2.877
Diastereomer 1c_7	2.718
Diastereomer 1c_8	2.493
Diastereomer 1c_9	2.435
Diastereomer 1c_10	2.298
Diastereomer 1c_11	2.244
Diastereomer 1c_12	2.227
Diastereomer 1c_13	2.171
Diastereomer 1c_14	1.65
Diastereomer 1c_15	1.32
Diastereomer 1c_16	1.082
Diastereomer 1c_17	0.997
Diastereomer 1c_18	0.931
Diastereomer 1c_19	0.867
Diastereomer 1c_20	0.797
Diastereomer 1c_21	0.759
Diastereomer 1d_1	9.986
Diastereomer 1d_2	9.897
Diastereomer 1d_3	7.193

Diastereomer 1d_4	7.068
Diastereomer 1d_5	6.824
Diastereomer 1d_6	5.628
Diastereomer 1d_7	3.856
Diastereomer 1d_8	3.29
Diastereomer 1d_9	3.212
Diastereomer 1d_10	1.884
Diastereomer 1d_11	1.867
Diastereomer 1d_12	1.854
Diastereomer 1d_13	1.747
Diastereomer 1d_14	1.712
Diastereomer 1d_15	1.668
Diastereomer 1d_16	1.57
Diastereomer 1d_17	1.511
Diastereomer 1d_18	1.343
Diastereomer 1d_19	1.136
Diastereomer 1d_20	1.133
Diastereomer 1d_21	1.108
Diastereomer 1d_22	1.07
Diastereomer 1d_23	1.039
Diastereomer 1d_24	0.9
Diastereomer 1d_25	0.861
Diastereomer 1d_26	0.851
Diastereomer 1d_27	0.843
Diastereomer 1d_28	0.808
Diastereomer 1d_29	0.805
Diastereomer 1d_30	0.797
Diastereomer 1d_31	0.773
Diastereomer 1d_32	0.756
Diastereomer 1d_33	0.732
Diastereomer 1d_34	0.723
Diastereomer 1d_35	0.658
Diastereomer 1d_36	0.651
Diastereomer 1d_37	0.63
Diastereomer 1d_38	0.613
Diastereomer 1d_39	0.58
Diastereomer 1d_40	0.576
Diastereomer 1d_41	0.569
Diastereomer 1d_42	0.503
Diastereomer 1d_43	0.497
Diastereomer 1d_44	0.48
Diastereomer 1d_45	0.452
Diastereomer 1d_46	0.429
Diastereomer 1d_47	0.388
Diastereomer 1d_48	0.362
Diastereomer 1d_49	0.355

Diastereomer 1d_50	0.331
Diastereomer 1d_51	0.303
Diastereomer 1d_52	0.301
Diastereomer 1d_53	0.297
Diastereomer 1d_54	0.297
Diastereomer 1d_55	0.276
Diastereomer 1d_56	0.274
Diastereomer 1d_57	0.269
Diastereomer 1d_58	0.266
Diastereomer 1d_59	0.262
Diastereomer 1d_60	0.246
Diastereomer 1d_61	0.243
Diastereomer 1d_62	0.242
Diastereomer 1d_63	0.237
Diastereomer 1d_64	0.221
Diastereomer 1d_65	0.212
Diastereomer 1d_66	0.207
Diastereomer 1d_67	0.202
Diastereomer 1d_68	0.2
Diastereomer 1d_69	0.191
Diastereomer 1d_70	0.185
Diastereomer 1d_71	0.184
Diastereomer 1d_72	0.183
Diastereomer 1d_73	0.181

Table S3. Experimental (Exp.) and calculated (Cal.) chemical shift values (CS, δ) of diastereomers (**a–d**) of **1** and svalbamide A (**1**).

No.	**Exp. CS of Svalbamide A (1)**	**Cal CS of diastereomer 1a**	**Cal CS of diastereomer 1b**	**Cal CS of diastereomer 1c**	**Cal CS of diastereomer 1d**
C-1	19.3	27.36	27.52	26.97	27.08
C-2	30.7	33.9	33.75	34.55	34.37
C-3	18.0	23.22	23.13	23.87	23.37
C-4	57.2	65.25	65.13	64.87	64.61
C-6	171.0	170.57	170.91	170.26	170.80
C-9	49.4	56.07	56.00	56.07	55.92
C-10	170.8	165.72	167.90	165.36	167.73
C-11	28.0	37.83	37.42	37.92	37.01
C-12	38.0	44.71	44.49	44.45	44.70
C-14	174.2	172.4	172.20	172.63	172.25
C-17	43.4	42.37	44.60	42.98	44.66
C-18	67.5	75.28	74.49	75.27	74.74
C-20	36.7	40.7	44.91	42.50	45.24
C-21	25.2	33.09	32.63	32.63	33.41
C-22	26.5	32.84	33.57	34.79	33.04
C-23	36.0	40.48	42.63	43.02	41.47
C-24	33.7	39.3	40.40	39.36	39.90
C-25	19.1	27.75	26.71	28.03	27.08
C-26	28.9	35.31	37.39	35.55	35.09
C-27	11.2	18.67	19.78	20.14	17.98
H-28	0.88	0.96	0.96	0.95	0.96
H-29	0.88	0.97	1.01	0.92	1.04
H-30	0.88	0.89	0.88	0.93	0.9
H-31	1.96	2.23	2.23	2.14	2.17
H-32	0.84	1.18	1.15	1.2	1.11
H-33	0.84	0.8	0.8	0.83	0.81
H-34	0.84	0.6	0.6	0.67	0.65
H-35	4.18	3.72	3.64	3.72	3.59
H-36	7.83	3.55	3.43	3.55	3.4
H-37	8.10	6.84	6.67	6.91	6.7
H-38	4.30	3.7	3.62	3.65	3.56
H-39	2.26	2.39	2.48	2.44	2.44
H-40	1.82	1.5	1.52	1.55	1.56
H-41	3.16	2.83	2.84	2.82	2.85
H-42	3.16	3.04	3.02	3	2.98
H-43	7.78	3.29	3.3	3.36	3.3
H-44	2.29	2.47	2.07	2.48	2.06
H-45	2.23	1.66	1.61	1.66	1.58
H-46	3.78	3.7	3.78	3.74	3.75
H-47	4.65	2.92	3.19	2.79	3.16
H-48	1.33	1.11	1.29	1.21	1.31
H-49	1.33	1.51	1.27	1.47	1.24
H-50	1.34	1.36	1.33	1.43	1.28
H-51	1.24	1.25	1.23	0.98	1.27
H-52	1.23	1.17	1.37	1.17	1.15
H-53	1.23	1.28	1.21	1.23	1.27

H-54	1.25	1.4	1.28	1.13	1.24
H-55	1.05	0.99	1.03	1.28	1.27
H-56	1.28	1.43	1.26	1.31	1.39
H-57	0.81	0.92	1.01	1.03	0.96
H-58	0.81	0.84	1.04	1.02	0.99
H-59	0.81	0.77	0.51	0.52	0.82
H-60	1.09	1.24	1.21	0.93	1.31
H-61	1.28	1.16	1.15	1.33	1.3
H-62	0.82	0.85	0.93	0.85	0.88
H-63	0.82	0.91	0.85	0.95	0.85
H-64	0.82	0.75	0.86	0.92	0.8
